# Treatment of Chronic Venous Ulcers With Heterologous Fibrin Sealant: A Phase I/II Clinical Trial

**DOI:** 10.3389/fimmu.2021.627541

**Published:** 2021-02-23

**Authors:** Luciana P. F. Abbade, Silvia Regina Catharino Sartori Barraviera, Maria Regina Cavariani Silvares, Ana Beatriz B. de C. O. Lima, Gabriela R. Haddad, Márcia A. N. Gatti, Natália Bronzatto Medolago, Márcia Tonin Rigotto Carneiro, Lucilene Delazari dos Santos, Rui Seabra Ferreira, Benedito Barraviera

**Affiliations:** ^1^ Department of Infectology, Dermatology, Imaging Diagnosis and Radiotherapy, Botucatu Medical School (FMB), São Paulo State University (UNESP – Univ Estadual Paulista), Botucatu, Brazil; ^2^ Graduate Program in Nursing, Botucatu Medical School (FMB), São Paulo State University (UNESP – Univ Estadual Paulista), Botucatu, Brazil; ^3^ Graduate Program in Clinical Research, Botucatu Medical School (FMB), São Paulo State University (UNESP – Univ Estadual Paulista), Botucatu, Brazil; ^4^ Graduate Program in Tropical Diseases, Botucatu Medical School (FMB), São Paulo State University (UNESP – Univ Estadual Paulista), Botucatu, Brazil; ^5^ Nursing School of Sagrado Coração University (UNISAGRADO), Bauru, Brazil; ^6^ Clinical Research Unit (UPECLIN), Botucatu Medical School, São Paulo State University (UNESP – Univ Estadual Paulista), Botucatu, Brazil; ^7^ Center for the Study of Venoms and Venomous Animals (CEVAP), São Paulo State University (UNESP – Univ Estadual Paulista), Botucatu, Brazil

**Keywords:** varicose ulcer, biological dressings, fibrin tissue adhesive, fibrin glue, fibrin sealant

## Abstract

**Background:**

Heterologous fibrin sealant (HFS) consists of a fibrinogen-rich cryoprecipitate extracted from *Bubalus bubalis* buffalo blood and a thrombin-like enzyme purified from *Crotalus durissus terrificus* snake venom. This study evaluated the safety and immunogenicity of HFS, estimated the best dose, and assessed its preliminary efficacy in the treatment of chronic venous ulcers (CVU).

**Methods:**

A phase I/II non-randomized, single-arm clinical trial was performed on 31 participants, accounting for a total of 69 active CVUs. All ulcers were treated with HFS, essential fatty acid, and Unna boot for 12 weeks. The outcomes assessed were: (1) primary safety, immunogenicity analyses, and confirmation of the lowest safe dose; (2) secondary promising efficacy by analyzing the healing process. Immunogenicity was evaluated using the serum-neutralizing (IgM and IgG) and non-neutralizing (IgA and IgE) antibody techniques against the product. The immuno-detection of IgE class antibodies was assessed using dot-blot assay before and at the end of treatment. Positive samples on dot-blot assays were subsequently analyzed by western blotting to verify the results.

**Results:**

No severe systemic adverse events related to the use of HFS were observed. Local adverse events potentially related to treatment include ulcer pain (52%), peri-ulcer maceration (16%), peri-ulcer pruritus (12%), critical colonization (8%), peri-ulcer eczema (4%), the opening of new ulcers (4%), and increased ulcerated area 4%). Neutralizing and non-neutralizing antibodies did not show significant deviations at any of the evaluated time points. Blot assays showed that all patients presented negative immunological reactions, either before or after treatment, with the thrombin-like enzyme component. In addition, two participants showed a positive immunological reaction to the cryoprecipitate component, while another two were positive before and during treatment. Regarding the secondary outcomes of preliminary efficacy, a total healing and significant reduction of the area was observed in 47.5 and 22%, respectively. A qualitative improvement was observed in the wound beds of unhealed ulcers.

**Conclusions:**

The investigational HFS bioproduct proved to be safe and non-immunogenic with a good preliminary efficacy for the treatment of CVU, according to the protocol and doses proposed. A multicentric phase III clinical trial will be necessary to verify these findings.

## Introduction

Chronic venous ulcers (CVUs) are responsible for approximately 70% of chronic ulcers of the lower limbs. They affect a large portion of the adult population, causing significant economic and social impacts and reducing the quality of life (1). The main clinical treatment used for the healing of CVUs is compression therapy, which diminishes the effects of chronic venous hypertension on the macro- and microcirculation (2). Furthermore, local treatment with compressive therapy is also essential, since it promotes the preparation of the ulcer bed, contributing favorably to the evolution of the healing process (3).

The pharmaceutical industry is continuously researching and developing new products for the treatment of CVUs. Thus, research studies range from the simplest coverage, namely solutions for hygiene and antisepsis, to the most complex types of dressings, which actively interfere in the various stages of healing (4). Among several substances used, fibrin sealant acts as a reference for topical use since it is a compatible biological and biodegradable material whose activities reproduce the final cascade blood coagulation stages, which are composed of fibrinogen and thrombin (5). In the presence of small amounts of calcium and factor XIII, thrombin converts fibrinogen into insoluble fibrin, forming a stable fibrin network. Commercial fibrin sealants are also supplemented with aprotinin, an antifibrinolytic agent, to slow down clot fibrinolysis (6, 7).

Fibrin sealant plays an important role in hemostasis and the healing process, given that the network of fibrin and the matrix of proteins associated with it not only promotes angiogenesis, collagen synthesis, and wound contraction, but also contributes to accelerated re-epithelization (8–10). By remaining in the ulcer bed for at least four days and totally degrading within 10 days, fibrin sealant is an excellent scaffold for incorporating and facilitating cell growth and the release of growth factors and antibiotics (11, 12).

Currently, there are three basic types of fibrin sealants: (1) autologous (obtained from the individual’s own thrombin and cryoprecipitate), (2) homologous (thrombin and cryoprecipitate obtained from “pool” of human plasma donors), and (3) heterologous (obtained from animal compounds). The amount of autologous fibrin sealant obtained from the individual’s own thrombin and cryoprecipitate is low, making it unfeasible for its use. Similarly, homologous fibrin sealants can promote the transmission of infectious or parasitic diseases, despite the precautions taken by manufacturers to reduce the risk of transmitting viruses (13, 14). The use of commercial sealants in the treatment of UVs, although very useful in healing, has the inconvenience of having a high cost, due to the prolonged time of use and the large amount that must be applied to cover each wound.

Since the 1990s, the Center for the Study of Venoms and Venomous Animals (CEVAP) in São Paulo State University (UNESP) (São Paulo, Brazil) has been researching and developing a new fibrin sealant. Through *in vitro* and *in vivo* studies using different applications and species, the heterologous fibrin sealant (HFS), which comprised of a fibrinogen-rich cryoprecipitate extracted from buffaloes blood and a thrombin-like enzyme (a serine protease named gyroxin) purified from the venom of South American rattlesnakes, has been standardized (15, 16). The lack of human products in the composition of this fibrin sealant eliminates the risk of transmission of infectious and parasitic diseases carried by human blood (15, 17). In addition to its sealant, hemostatic, and adhesive functions, this new bioproduct also provides an excellent scaffold for stem cells (18, 19) and can be used as a drug delivery system (20, 21).

Previous HFS studies have demonstrated its low toxicity and non-mutagenicity (19), combined with preclinical studies in animals (15, 17) and preliminary studies in humans (22, 23), encouraging the use of this bioproduct in phase I/II clinical trials. In light of these favorable results, the primary objective of the present study was to evaluate the safety of the bioproduct: (i) by clinical evolution, laboratory exam results, and the existence of local and systemic adverse events; (ii) by the analysis of its immunogenicity; (iii) to estimate the lowest safe dose. As secondary objectives, this study evaluated the preliminary efficacy of the bioproduct by analyzing the evolution of the healing process and wound epithelization, and evaluating its capacity to diminish the ulcerated area and prepare the wound bed.

## Patients and Methods

### Study Design and Regulatory Agencies

A phase I/II, non-randomized, single-arm clinical trial on participants with active CVUs was carried out between September 2015 and March 2017.

The clinical protocol was approved in advance by the National Commission on Ethics in Research (CONEP) (Certificate of Presentation of Ethical Appreciation no. 37410814.4.0000.5411) (v8, approved on 12/16/2014) and the National Health Surveillance Agency (ANVISA), whose Consent Record of Sealant Study II was approved on 06/05/2015 (no. 0498468154) (proc. no. 25351010938201571). This trial was registered on 11/11/2014 in the Brazilian Clinical Trials Registry (ReBEC) at http://www.ensaiosclinicos.gov.br/. The first participant was enrolled on 09/09/2015 (Universal Trial Number (UTN) U1111-1163-9824 and register number RBR-9mbdj3). The public access URL is available at http://www.ensaiosclinicos.gov.br/rg/RBR-9j7qqr/. The participants originated from the Chronic Ulcers Ambulatory Unit at the Dermatology Service of the Hospital of Clinics at the Botucatu Medical School, São Paulo State University (UNESP) (São Paulo, Brazil) and were treated at the Clinical Research Unit (UPECLIN) of the same university. All participants consented to participate by signing the terms of free and informed consent (TFIC).

The data from all the participants were collected by five physician researchers (dermatologists) (LPFA, SRCS, MRCS, ABCOL, and GRH), and the data were registered and stored in an electronic formulary (Eletronic Case Report Form (eCRF)) developed specifically for this study (http://www.crf.fmb.unesp.br/selante/login.php?accesscheck=%2Fselante%2Findex.php) by the support team at UPECLIN. eCRF generated spreadsheets in Excel format, which were used for statistical analysis.

### Eligibility Criteria

The participants who met the eligibility criteria for the study were treated with HFS, essential fatty acid, and Unna boot for 84 days. Thirty-one participants, of both sexes, with chronic ulcers of venous etiology in the lower limbs were selected for the study, provided they met the inclusion and exclusion criteria described below.

#### Inclusion Criteria

- Sign the terms of free and informed consent (TFIC);
- Be at least 18 years of age, for both sexes;
- Have chronic venous disease with CVU evidenced by one or more of the following signs:
  - Hyperpigmentation of the distal third of a lower limb;
  - Stasis eczema;
  - Lipodermatosclerosis;
  - Varicose veins.
- Have at least one ulcer, whose evolution time is between a minimum of 6 weeks and a maximum of 5 years;
- Present a sum of ulcer areas of both limbs between 2 and 60 cm^2^;
- Have at least one ulcer with an area greater than 2 cm²;
- The following medications were not taken within the two weeks prior to the initiation of treatment:
  - Venotonics;
  - Pentoxifylline;
  - Fibrinolytic drugs.
- Be available to attend the UPECLIN once per week to complete the treatment.

#### Exclusion Criteria

- Have ulcers in the lower limbs from other etiologies (hematological, neoplastic, infectious, or other causes);
- Take anticoagulants;
- Have an infected ulcer, that is, associated with erysipelas, cellulitis, or lymphangitis
- Have an ulcer with critical colonization, that is, a large quantity of exudation and/or a fetid odor and/or bed staining/wound coloration yellowish and/or greenish and/or opaque red and/or tissue of friable granulation.
- Present necrosis in the ulcer bed;
- Have an ulcer with a devitalized tissue covering the entire bed;
- Have venous ulcer associated with peripheral arterial disease characterized as an ankle brachial index (ABI) < 0.9;
- Being unable or unwilling to adhere to compressive treatment of the lower limb for seven days;
- Have a prior history of allergy to Unna boot treatment;
- Have a prior history of allergy to treatment with essential fatty acid;
- Have suspicion or confirmation of pregnancy;
- Have coagulogram values outside the limits of normality (TTPA >1.25 and prothrombin activity time <70% or >100%)
- Be a woman of fertile age not utilizing secure contraceptive methods.

#### Observations:

1. Participants with an infected ulcer, with critical colonization, with the presence of necrosis, and with devitalized tissue covering the entire bed could be included after the adequate treatment of these conditions.
2. Women of fertile age who do not use secure contraceptive methods could be included if they agreed to the use of at least one reliable contraceptive method.
3. Participants could only be included once. Those who participated in the study and had their ulcers healed were not eligible for this study again if there were recurrences.

#### Discontinuation Criteria

After inclusion in the study, participation was discontinued if the individual:

- Removed or withdrew from the terms of consent (TFIC);
- Initiated the use of an anticoagulant during the study;
- Presented coagulogram values outside the limits of normality (TTPA >1.25 and prothrombin activation time <70 or >100%) with clinical significance.
- Presented infection associated with ulcer(s) (erysipelas, cellulitis, or lymphangitis) during follow-up. In these cases, the participant was included in the routine treatment of the dermatology service.
- Had critical colonization during the follow-up, according to the researcher’s discretion. The only procedure allowed for the treatment of colonization was superficial surgical debridement, without local anesthetic, for a maximum of three times during the study and before the dressing was applied. If there was a need for topical and/or systemic antibiotics, participation was terminated;
- Utilized other treatments not recommended in the protocol;
- Removed the Unna boot within less than five days after its application;
- Became pregnant;
- Had distal pulses not palpable and ABI < 0.9;
- Presented a severe adverse event, according to the researcher’s discretion;
- Presented clinically significant local adverse events, such as severe pain, eczema, enlarged area, or the opening of a new ulcer, at the researcher’s discretion.

### Production of Investigational Product (HFS)

#### Purification of Thrombin Like-Enzyme

CEVAP maintains a serpentarium for the breeding and milking of specimens with authorization and registration as a scientific breeder for research purposes at the Brazilian Institute of the Environment and Natural Resources (IBAMA) (protocol number 02001.005670/90–77), in addition to having authorization for the management of wild fauna (no. 3507.7263/2012-SP). Standard operational protocols (SOPs) are followed rigidly in environments where *Crotalus durissus terrificus* snakes are housed for venom production, according to the international good management practices to ensure the quality and purity required for the production of biopharmaceuticals (17, 24). Further information on CEVAP is provided at https://youtu.be/CPcs4ity-Uw.

First, the venom was extracted and filtered. Then, the protein dosage was evaluated, followed by lyophilization and storage in a refrigerator between +4 and +8°C. Subsequently, the venom was subjected to fractionation *via* high-performance liquid chromatography (HPLC). Lastly, the purity of the thrombin-like enzyme (serine protease, gyroxin) component was evaluated using sequencing and mass spectrometry as described by Barros et al. (25). Further information on HFS is provided at https://youtu.be/y6ho6M0amA8.

#### Extraction and Processing of Cryoprecipitate

*Bubalus bubalis* buffaloes are ideal for the large-scale production of cryoprecipitate (11, 15). Specimens housed at Céu Azul Farm, located in the municipality of Pereiras (São Paulo, Brazil) were kindly provided by Mr. Aristides Pavan. CEVAP researchers carried out sanitary management practices on a monthly basis, including vaccinations, deworming, isolation and quarantine when necessary, the protection of animals against vectors of infectious diseases, diagnostic serological tests against zoonoses, and an annual evaluation of the hypersensitivity test against tuberculosis (Mantoux or PPD), in addition to clinical examinations performed by a veterinarian. These actions are recommended by the Secretary of Agriculture and Supply for the State of São Paulo by the Department of Animal Health of the Secretary of Agricultural Defense of the Ministry of Agriculture, Livestock, and Supply (MAPA), Brazil, and by the World Health Organization (WHO), and were conducted under the coordination of the Guilherme Shin Iwamoto Haga (CRMV/SP 19621) veterinarian. All animals were microchipped in order to maintain the biosafety and traceability of the extracted cryoprecipitate. The academic bases for processing and traceability were published by Pontes et al. (26) and Ferreira Jr. et al. (27). Details of the formulation are protected by patents no. BR 10 2014 011432 7 and BR 10 2014 011436-0 (28, 29).

#### Formulation of the Product

**Figure 1:**
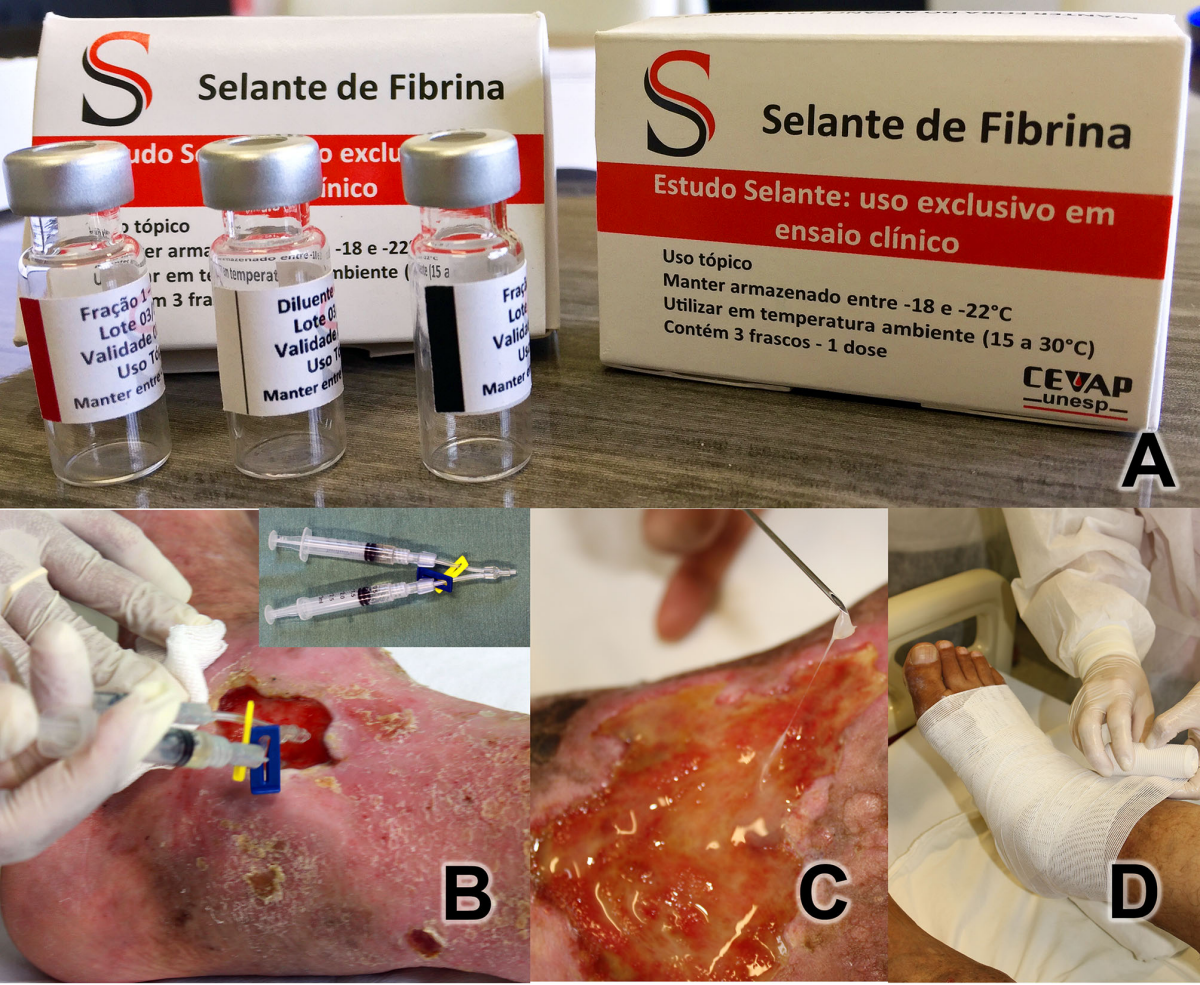
**(A)** Heterologous Fibrin Sealant Package with the three bottles. Diluent vial (white stripe) containing 0.6 mL of calcium chloride; Fraction 1 vial (red stripe) containing 0.4 mL of serine protease extracted from the venom of *Crotalus durissus terrificus*; Fraction 2 vial (black stripe) containing 1 mL of cryoprecipitate rich in fibrinogen and coagulation factors extracted from buffaloes. **(B)** Application investigational product on the ulcerated bed. Y-connector using two syringes (in detail) with 1 mL of components each one. **(C)** Detail of fibrin adhered to the ulcer. **(D)** After covering the ulcer bed with gauze soaked in essential fatty acids, the Unna boot was applied.

Each dose of the medication for topical use was packaged and distributed in three bottles (**Figure 1A**): (1) diluent vial containing 0.6 mL of calcium chloride (white stripe); (2) component 1 vial containing 0.4 mL of the thrombin-like enzyme (red stripe); (3) component vial 2 containing 1 mL of cryoprecipitate (black stripe). All vials were stored in a freezer at ‒20°C until use. The composition of the product is described in detail in patents no. BR 10 2014 011432 7 and BR 10 2014 011436-0 (28, 29).

#### Application of Product in Ulcers

The product was applied to each participant once a week for 12 weeks. Prior to application, packages containing the three vials were thawed by removing from the freezer 15 min before application. After cleaning the wounds with 0.9% physiological saline, the product was applied to the ulcerated areas.

The diluent vial component (white stripe, 0.6 mL) was mixed into a fraction 1 vial (red stripe, 0.4 mL) and taken up in a 1-mL syringe. The fraction 2 vial (black stripe: 1 mL) was used to fill a second 1-mL syringe. Both syringes were connected to a duo needle-free valve extension line (Y-connector) (**Figure 1B**). Then, the contents of the two syringes were applied concurrently over the entire bed of the ulcer, mixing the two HFS components. Total polymerization was expected to occur for approximately 3–5 min, ultimately appearing as a colorless gel (**Figure 1C**). Afterwards, gauzes containing essential fatty acids were placed in a sufficient quantity to cover the ulcerated surface, and the dressing was finished with an Unna boot bandaging half and half, from the foot to immediately below the knee (**Figure 1D**). Participant were instructed to not remove the dressing and to wear it for seven days until their return visit to the clinic.

### Outcomes

The outcomes were classified into primary and secondary outcomes.

#### Primary Outcomes

These outcomes were related to the safety and confirmation of the proposed dose.

- Evaluation of local adverse events (AEs): pain, itching, maceration of the ulcer edge, eczema, local infection, critical colonization, increased ulcer extension, and opening of a new ulcer.
- Evaluation of systemic AEs: potential blood clotting disorders in addition to clinical systemic alterations.
- Evaluation of laboratory alterations: Complete blood count, erythrocyte sedimentation rate (ESR), prothrombin time (PT), activated partial thromboplastin time (APTT), and fibrinogen and C-reactive protein (CRP) were determined according to the protocol proposed and approved by the Ethics Commission.
- The minimum necessary dose of the investigational product to cover a maximum ulcer surface area of 60 cm^2^ per participant was evaluated and confirmed, as proposed in the clinical protocol.
- Immunogenicity: performed using nephelometry, dot blot, and western blotting. For this purpose, blood was collected from all participants; the serum was aliquoted and stored at −80°C until the exams were performed.

The AEs were classified according to the guidelines of the International Conference for Harmonization (ICH) (30), which considers AE to be any undesired clinical occurrence in a clinical research participant, who receives a pharmaceutical product that does not necessarily have a causal relationship with this treatment. It is worth mentioning that AE registration was performed over 12 weeks of follow-up. Thus, an AE can be any unfavorable and unintended sign (including an abnormal laboratory finding, symptoms, or disease temporally associated with the use of a product under investigation, considered to be related or not to it). Pre-existing conditions that worsened during the study were also reported as AEs. Any changes in laboratory tests were considered systemic AE as well as systemic changes detected by clinical history (primary outcomes).

The causal relationship between the investigational product and AE was determined by the researchers’ judgment at the time of clinical care and classified as:

- Probable: The temporal relationship is well defined and there is no other possible causal factor. In this case, there is an almost certain relationship between the reaction and the medication.
- Possible: The temporal relationship between the event and medication administration is well defined, but there is another possible causal factor.
- Remote: The relationship with the drug is unlikely, but it cannot be definitively ruled out.
- Not related: The temporal relationship between the event and the ingestion or administration of the medication is non-existent or doubtful or, yet, there is another factor that can respond as a causal factor of the reaction.

### Nephelometry, Dot Blot, and Western Blotting

Using nephelometry, the total IgA, IgE, IgG, and IgM types were dosed at zero time (T0 – screening), that is, before the initiation of treatment, and again at T1 (30 days), T2 (60 days) after the start of treatment, and T3 (90 days) at the end of treatment (31). Furthermore, the antibodies of the specific IgE class, which would have been produced by the patients against the cryoprecipitate and/or against the thrombin-like enzyme (serine protease), the main sealant components, were investigated in the serum before the beginning and at the end of the treatment. Thus, a potential turning point and sensitization of participants to the investigational product was sought. To this end, the dot blot assay was performed according to the immunodetection procedures proposed by Santos et al. (32). Samples that were positive in dot blot tests were subsequently subjected to western blotting tests for structural identification of the allergen responsible for sensitization (32). In the dot blot assay, 4 µL of each component (cryoprecipitate or thrombin-like enzyme – serine protease) was immobilized on a 2 × 2 cm nitrocellulose membrane. Once immobilized, they were incubated with the participants’ serum. In this case, those who eventually developed antibodies and were therefore sensitized to the evaluated components will contain specific antibodies in their blood that will appear on the mentioned nitrocellulose membrane. From this stage, the use of secondary anti-IgE antibody (Abcam) followed by the streptavidin conjugate (HRP) (Abcam) was able to signal which patients were sensitized and developed immunogenicity against the components of the sealant.

#### Secondary Outcomes

These were evaluated according to the preliminary effectiveness of the product by monitoring the evolution of the ulcerated area (diminished area and complete healing or reepithelization) of the evolutionary ulcer-bed characteristics (decrease or absence of devitalized tissues in the ulcer bed during treatment) and of diminution in the exudation quantity.

### Statistical Considerations

The sample size was estimated considering examples of phase I/II clinical studies, which aim to evaluate safety and confirm the minimum application dose required. The failure to carry out this study on 10 participants, as recommended in the phase I trials, is justified by the fact that the efficacy outcome is one of the targets, although there are, to date, no data on safety, nor confirmation of an adequate dose for the product under investigation.

For statistical analysis, descriptive summaries and confidence intervals were calculated, and McNemar’s test was applied to analyze the evolution of ulcers before and at the end of treatment. Categorical variables are presented as percentages. Continuous variables were assessed for normality using the Shapiro–Wilk test and presented as the means and standard deviation (SD) or the median and quartiles (p25–p75), if indicated.

A significance level of 5% was considered for the analysis, performed using the software SPSS v21.0 (33). Product safety was assessed by observing local and systemic adverse events, in addition to clinical and laboratory parameters.

## Results

### General Aspects

From September 2015 to March 2017, 31 of the 40 participants evaluated met the eligibility criteria and were included, accounting for a total of 69 active CVUs. Sixteen were male (51.6%); the mean age was 65.9 (± 14.0) years; and the main antecedent was systemic arterial hypertension (61.3%), as shown in **Tables 1** and **2**.

**Table 1:** General characteristics of the 31 participants included in the study at baseline.

Identification (initials and N° in the study)	Gender	age	BMI	SAH	DM	DVT	Total number of ulcers	Initial area (cm^2^)
ALC16	M	37	34.6	No	No	Yes	3	44.37
AMB03	M	53	26.7	No	No	Yes	2	14.05
BP26	M	52	29.1	No	No	Yes	5	21.99
C-C36	F	67	32.0	Yes	Yes	No	1	6.36
CA23	M	44	27.3	No	No	No	1	17.39
CL12	F	64	32.8	Yes	No	No	2	11.04
CRS33	M	59	36.8	No	No	Yes	5	19.77
E-S01	F	78	24.7	Yes	Yes	No	2	17.27
EEV13	F	83	16.9	No	No	No	2	3.99
FAS05	M	63	26.4	Yes	Yes	Yes	3	9.54
HFS28	F	74	30.3	Yes	No	No	2	47.42
IMS35	F	89	23.9	Yes	No	No	4	28.77
J-M39	M	66	28.5	No	No	No	1	2.01
J-S42	M	86	33.5	Yes	No	Yes	1	51.83
JPF18	M	55	21.8	No	No	Yes	1	11.19
JSF29	M	84	27.8	Yes	No	No	1	15.12
LOF22	M	56	32.4	No	Yes	No	1	14.87
LS15	M	78	29.8	Yes	Yes	No	2	15.54
MIV14	F	70	38.3	Yes	Yes	No	6	38.50
MPG17	F	78	29.7	Yes	Yes	No	3	58.45
NMA30	F	78	30.9	Yes	No	No	2	4.03
NS07	F	73	41.2	Yes	Yes	No	2	52.90
PLB27	F	42	52.2	No	No	No	2	11.37
SVR04	F	65	31.4	Yes	No	No	2	20.66
TAA10	F	87	21.2	Yes	No	No	4	9.61
VAR21	M	63	31.5	Yes	No	No	1	60.00
VAT34	F	51	40.4	No	No	Yes	1	2.93
VD19	M	49	25.1	No	No	No	1	36.43
VDR32	M	68	25.4	Yes	No	No	2	41.15
VLA11	F	75	48.2	Yes	Yes	No	2	7.60
WN37	M	58	26.1	Yes	No	No	2	4.40

M, male; F, female; BMI, body mass index; DM, diabetes mellitus; SAH, systemic arterial hypertension; DVT, deep vein thrombosis.

Identification (initials and No. in the study).

**Table 2:** Baseline clinical characteristics of the 31 participants included in the study and the 25 participants who were followed for 12 weeks.

Variables	All participants included (n=31)	Participants who completed the study (n=25)
**Mean age (± SD) in years**	65.9 (14.0)	66.0 (14.8)
**Mean BMI (± SD)**	30.9 (7.5)	30.9 (6.0)
**Female n (%)**	15 (48.4)	11 (44.0)
**Male n (%)**	16 (51.6)	14 (56.0)
**SAH n (%)**	19 (61.3)	15 (60.0)
**DM n (%)**	09 (29.0)	09 (36.0)
**Smoking history n (%)**	03 (9.7)	2.0 (8.0)
**History of lower limb DVT n (%)**	08 (25.8)	08 (32.0)

BMI, body mass index; SAH, systemic arterial hypertension; DM, diabetes mellitus; DVT, deep vein thrombosis.

**Figure 2:**
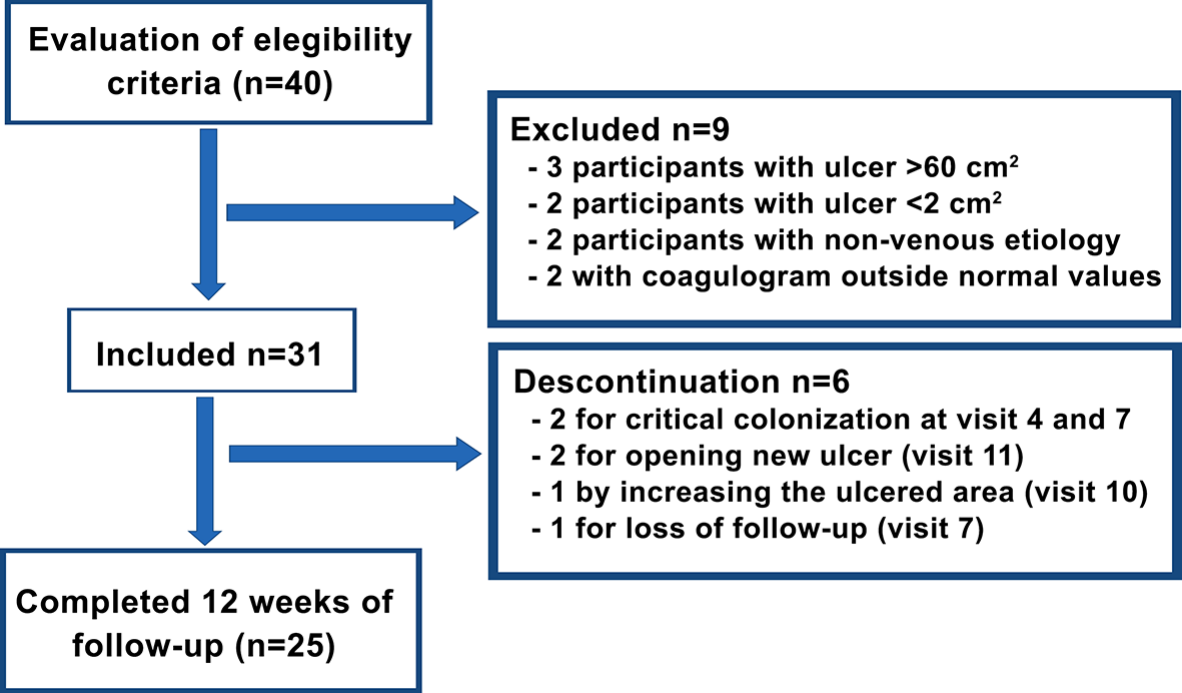
Study flowchart.

**Figure 2** shows the study flowchart. Six participants were discontinued from the study (19.3%) for the following reasons: critical colonization of the ulcer in two participants (6.45%), opening of new ulcers in two participants (6.45%), an increase in the ulcerated area in one participant (3.22%), and loss of follow-up in one participant (3.22%), although his ulcer was practically healed. Therefore, 25 participants completed 12 weeks of follow-up.

The clinical characteristics of all evaluated ulcers are described in **Table 3**. A similar distribution was observed between the limbs and preferential location in the medial region of the leg, with a mean active ulcer time of 1.8 years (± 2.0).

**Table 3:** Distribution of ulcers from all participants included and from those who completed the study according to their clinical characteristics at baseline.

Variables	All participants included (n=31 participants with 69 ulcers)	Participants who completed the study (n=25 participants with 59 ulcers)
**Affected lower limb**		
-Left	35 (50.7%)	30 (50.8)
-Right	34 (49.3%)	29 (49.2)
**Localization**		
- Medial region of the leg	38 (55.1%)	35 (59.3)
- Lateral region of the leg	12 (17.4%)	12 (20.3)
- Anterior region of the leg	4 (5.8%)	4 (6.8)
- Posterior region of the leg	1 (1.4%)	0 (0.0)
- Dorsal aspect of the foot	4 (5.8%)	2 (3.4)
- Medial aspect of the foot	6 (8.7%)	4 (6.8)
- Lateral aspect of the foot	5 (7.2%)	2 (3.4)
**Active ulcer (years) median (p25-p75)**	0.7 (0.3 – 3)	0.7 (0.3 – 3)
**Pain (VAS) median (p25-p75)**	3.0 (0.0 – 7.0)	3.0 (0.0 – 6.0)
**Ulcer area (cm^2^) median (p25-p75)**	4.1 (2.0 – 12.7)	4.1 (2.0 – 12.6)

VAS, visual analog scale.

### Primary Outcomes

#### Safety—Adverse Events

Regarding the AEs of the 31 participants included in the study, 85 were observed, of which 61 were considered local and 24 were systemic. Nineteen participants (61.3%) had more than one EA, and nine participants (29%) also had local and systemic EAs. Most AEs were mild or moderate, with only one having pain classified as severe AE. Of the total local AEs, 25 [25/61 (40.1%)] were classified as possibly related to the investigational product (**Table 4**).

**Table 4:** List of local adverse events possibly related to the product.

EA possibly related to the product	Number of participants (n=31)n (%)	Number of AE* possibly related to the product(n=25) n (%)	Intensity of AE		
			Mildn (%)	Moderaten (%)	Severen (%)
Ulcer pain	08 (25.8)	13 (52.0)	01 (7.7)	11 (84.6)	01 (7.7)
Periulcerous maceration	04 (12.9)	04 (16.0)	03 (75.0)	01 (25.0)	0 (0.0)
Periulcerous itching	03 (9.7)	03 (12.0)	02 (66.6)	01 (33.3)	0 (0.0)
Critical colonization	02 (6.4)	02 (8.0)	01 (50.0)	01 (50.0)	0 (0.0)
Periulcerous eczema	01 (3.2)	01 (4.0)	0 (0.0)	01 (100.0)	0 (0.0)
Opening of a new ulcer	01 (3.2)	01 (4.0)	01 (100.0)	0 (0.0)	0 (0.0)
Increase in the ulcerated area	01 (3.2)	01 (4.0)	01 (100.0)	0 (0.0)	0 (0.0)

*The number of adverse events (AE) may differ from the number of participants with AE, as the same participant can have more than one episode of the same EA.

There were 24 systemic AEs detected by laboratory changes, including eosinophilia (one participant), or by medical history, such as arterial hypertension (two participants), sinusitis (two participants), lower back pain (two participants), epistaxis (four events in the same participant), cough (two events in the same participant), epidermoid cyst infection (one participant), acute kidney injury (one participant), headache (one participant), facial paralysis (one participant), diffuse eczema (one participant and related to concomitant moisturizing lotion), cold (one participant), nausea and vomiting (one participant), hypoglycemia (one participant and related to concomitant hypoglycemic medication), chest pain (one participant), and dry mouth (one participant). None were attributable to the product.

There was no statistically significant difference between the averages before and throughout the treatment of subsidiary laboratory tests, such as complete blood count, ESR, VT, TPT, fibrinogen, and CRP (**Supplementary Material 1**).

#### Safety—Immunogenicity

The serum levels of IgM, IgG, IgA, and total IgE in 30 participants (the material collected from one participant was mislaid) were determined before, during, and at the end of treatment, the results of which are described in **Tables 5**–**8**.

**Table 5:** Evaluation of the plasma concentration of Immunoglobulin M (IgM) in mg/dL of 30 participants^#^ at T0, T1, T2 and T3.

Participants	T0	T1	T2	T3
ALC16	118	103	113	118
AMB03	128	110	130	*
BP26	55	50	42	51
C-A23	95	84	96	*
C-C36	89	91	87	88
CL12	74	94	83	69
CRS33	94	107	111	109
E-S01	106	86	86	100
EEV13	124	133	128	119
FAS05	40	38	42	**
HFS28	155	147	**	**
IMS35	58	55	55	54
J-S42	60	49	60	50
JPF18	109	107	84	80
JSF29	53	53	**	**
LS15	65	67	57	60
LOF29	39	41	42	42
MIV14	60	66	63	60
MPG17	110	103	104	100
NS07	84	80	93	85
NMA30	172	165	158	**
PBL27	116	106	111	**
SVR04	59	62	59	**
TAA10	83	84	76	82
VD10	173	173	176	168
VAR21	156	138	151	168
VAT34	84	85	89	80
VDR32	83	82	67	68
VLA11	64	79	79	72
WN37	73	89	90	*

T0 before treatment (screening); T1 (30 days), T2 (60 days) during treatment, and T3 (90 days) at the end of treatment.

*Participants who did not collected material for immunogenicity analyses at that visit.

**Discontinued participants.

IgM references values for adult individuals, 50 to 300 mg/dL.

^#^Mislaid of material collected for immunogenicity analyzes from all visits by one participant.

**Table 6:** Evaluation of the plasma concentration of Immunoglobulin G (IgG) in mg/dL of 30 participants^#^ at T0, T1, T2, and T3.

Participants	T0	T1	T2	T3
ALC16	1.289	1.234	1.199	1.227
AMB03	1.254	1.210	1.230	*
BP26	1.164	1.166	1.005	1.034
C-A23	1.248	1.301	1.211	*
C-C36	1.741	1.720	1.507	1.886
CL12	1.448	1.498	1.222	1.261
CRS33	1.380	1.320	1.396	1.369
E-S01	1.255	1.014	1.040	1.153
EEV13	1.094	1.185	1.072	1.025
FAS05	1.013	1.021	1.262	**
HFS28	1.136	1.105	**	**
IMS35	1.881	1.895	2.047	2.097
J-S42	1.781	1.663	1.552	1.743
JPF18	1.770	1.095	1.680	1.405
JSF29	1.423	1.531	**	**
LS15	1.423	1.420	1.213	1.338
LOF29	1.458	1.403	1.304	1.590
MIV14	1.429	1.354	1.198	897
MPG17	1.143	1.098	1.074	1.130
NS07	1.607	1.485	1.565	1.460
NMA30	1.371	1.541	1.388	**
PBL27	1.718	1.636	1.820	**
SVR04	1.312	1.181	1.203	**
TAA10	1.211	1.164	1.093	1.137
VD19	1.247	1.189	1.177	1.164
VAR21	1.407	1.120	1.248	1.354
VAT34	1.192	1.138	1.217	1.034
VDR32	1.664	1.641	1.698	1.680
VLA11	1.278	1.234	1.154	1.110
WN37	1.047	1.179	1.126	*

T0 before treatment (screening); T1 (30 days), T2 (60 days) during treatment, and T3 (90 days) at the end of treatment.

*Participants who did not collected material for immunogenicity analyses at that visit.

**Discontinued participants.

IgG references values for adult individuals, 490 to 1.140 mg/dL.

^#^Mislaid of material collected for immunogenicity analyzes from all visits by one participant.

**Table 7:** Evaluation of the plasma concentration of Immunoglobulin A (IgA) in mg/dL of 30 participants^#^ at T0, T1,T2, and T3.

Participants	Gender	T0	T1	T2	T3
ALC16	M	622	687	679	657
AMB03	M	434	409	434	*
BP26	M	370	384	333	332
C-A23	F	181	157	188	*
C-C36	M	385	416	402	431
CL12	F	244	276	216	214
CRS33	M	209	210	203	212
E-S01	F	732	500	531	516
EEV13	F	479	524	499	460
FAS05	M	224	215	247	**
HFS28	F	252	255	**	**
IMS35	F	425	423	423	415
J-S42	M	719	476	472	468
JPF18	M	<40	370	<40	<40
JSF29	M	253	267	**	**
LS15	M	472	469	460	533
LOF29	M	259	274	268	273
MIV14	F	406	411	409	330
MPG17	F	599	540	564	589
NS07	F	584	613	641	610
NMA30	F	313	343	313	**
PBL27	F	220	213	212	**
SVR04	F	267	249	242	**
TAA10	F	364	371	335	342
VD19	M	140	134	142	127
VAR21	F	532	445	483	513
VAT34	M	152	144	154	134
VDR32	M	266	295	253	252
VLA11	F	168	195	185	174
WN37	M	229	267	272	*

T0 before treatment (screening); T1 (30 days), T2 (60 days) during treatment, and T3 (90 days) at the end of treatment.

*Participants who did not collected material for immunogenicity analyses at that visit.

**Discontinued participants.

IgA references values for adult individuals, Male, 83.0 to 406.0 mg/dl; Female, 70.0 to 374.0 mg/dL.

^#^Mislaid of material collected for immunogenicity analyzes from all visits by one participant.

**Table 8:** Evaluation of the plasma concentration of Immunoglobulin E (IgE) in mg/dL of 30 participants^#^ at T0, T1,T2, and T3.

Participants	T0	T1	T2	T3
ALC16	1.751	1.803	1.682	2.178
AMB03	792	741	699	*
BP26	16.725	15.642	12.703	10.748
C-A23	7.998	6.123	5.904	*
C-C36	1.982	2.317	2.168	2.100
CL12	327	359	321	348
CRS33	937	811	572	822
E-S01	7.953	6.625	8.664	6.167
EEV13	111	115	76	94
FAS05	3.026	2.847	3.929	**
HFS28	1.393	1.400	**	**
IMS35	4.621	4.633	5.177	5.571
J-S42	29.758	15.186	14.265	15.187
JPF18	28	1.826	35	567
JSF29	2.931	3.374	**	**
LS15	1.375	1.494	3.227	261
LOF29	25	25	25	25
MIV14	921	686	474	364
MPG17	518	204	677	899
NS07	1.817	3.405	4.759	4.777
NMA30	1.742	1.306	954	**
PBL27	1.550	1.655	1.979	**
SVR04	209	239	315	**
TAA10	1.123	1.205	916	957
VD19	807	657	833	720
VAR21	173	177	143	207
VAT34	555	25	25	526
VDR32	1.994	1.743	1.635	1.684
VLA11	4.889	5.461	4.995	4.145
WN37	715	929	1.174	*

T0 before treatment (screening); T1 (30 days), T2 (60 days) during treatment, and T3 (90 days) at the end of treatment.

*Participants who did not collected material for immunogenicity analyses at that visit.

**Discontinued participants.

IgE references values for adult individuals, 1 to 183 UL/mL.

^#^Mislaid of material collected for immunogenicity analyzes from all visits by one participant.

The serum levels of IgM and total IgA were unchanged throughout the treatment with the product. As to the IgG levels, only four participants [EEV13, FAS05, HFS28, and WN37 (**Table 6**)] presented normal levels before treatment. Most of the participants showed slight changes in the levels of this immunoglobulin across the treatment, sometimes decreasing, sometimes increasing. As for IgE (**Table 8**), during treatment, the levels were significantly elevated in eight participants (ALC16, C-C36, IMS35, JPF18, MPG17, NS07, PBL27, and WN37), although these already showed high pretreatment levels.

**Figure 3:**
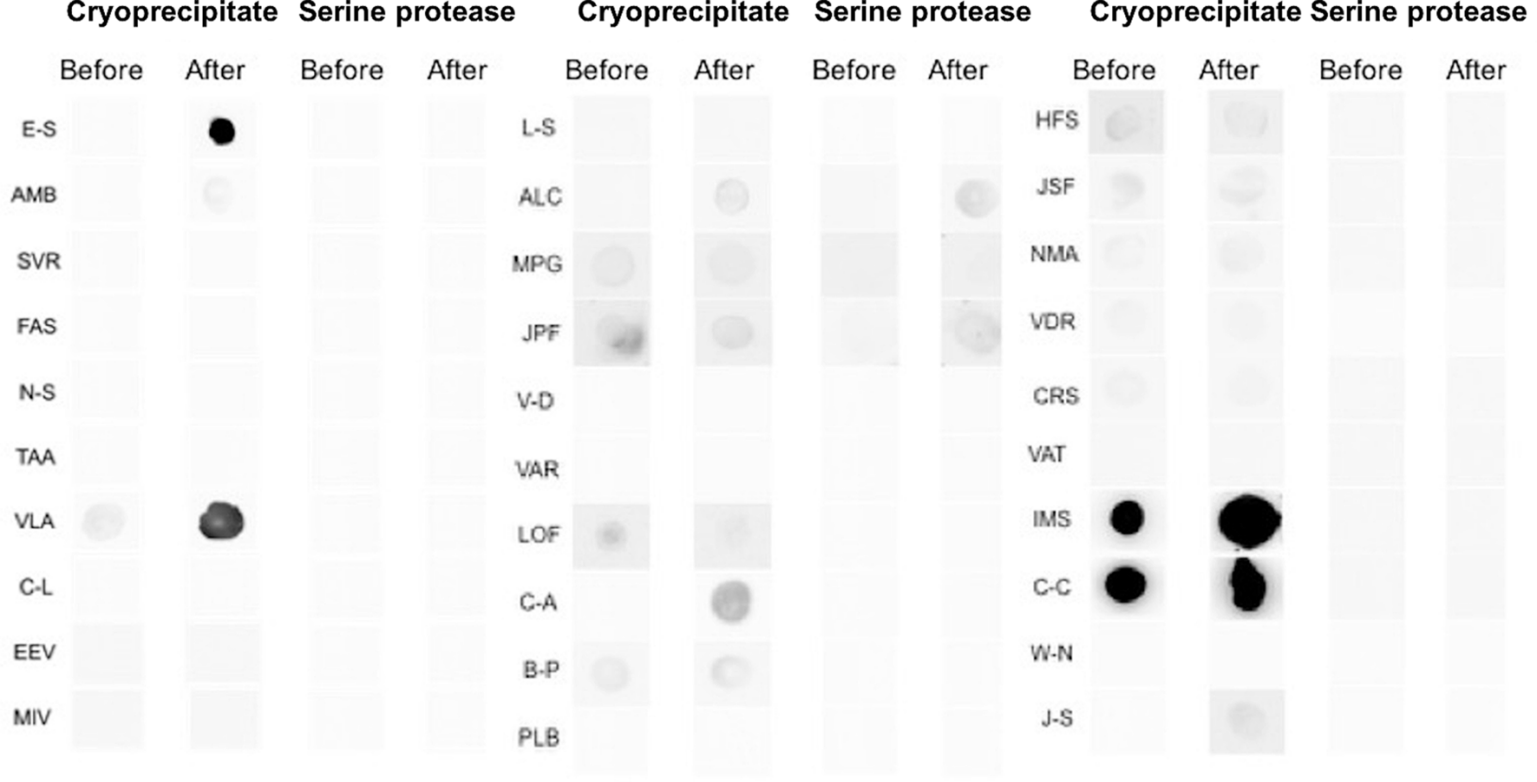
Immunogenicity analysis using the secondary anti-IgE antibody (Abcam) followed by the streptavidin conjugate [HRP] (Abcam) to capture antibodies against cryoprecipitate and serine protease (thrombin like-enzyme) present in the serum of patients treated with heterologous fibrin sealant. The letters (left side of the image) correspond to the initials of the names of the study participants. The data are for 30 participants and not 31, as there was a mislaid of material collected for immunogenicity analysis from all visits by one participant.

The results of the dot blot test are displayed in **Figure 3**. The participants’ sera before (screening) and during treatment with the investigational product were challenged against the cryoprecipitate and thrombin-like enzyme, the main components of the bioproduct. None of the patients had a positive reaction neither before nor at the end of treatment to thrombin-like enzyme. In relation to cryoprecipitate, four participants were positive at the conclusion of treatment. Of these four, two were negative before and positive at the end, and two were already positive and remained positive at the end of the treatment.

To evaluate the safety of the product in relation to immunogenicity, we compared the data observed in nephelometry and dot blot with the clinical aspects of local AEs of greater relevance, such as enlargement of the ulcerated area, the opening of new ulcers, and peri-ulcer eczema. The opening of a new ulcer was not found to be related to the product, given that of the four participants who had this AE, only one presented a reaction in the dot blot to cryoprecipitate on the 10th visit. An increase in the ulcerated area occurred in three participants, and this AE was also unrelated to the use of the bioproduct, as none of the participants showed a change in the dot blot reaction. The appearance of peri-ulcer eczema occurred in two participants and possibly was not related to the bioproduct, since only one of the participants presented a positive dot blot reaction before treatment, thus remaining the same at the end. Two participants had a positive reaction on the dot blot at the 10th visit (E-S and VLA (**Figure 3**), although both had a good evolution of the healing process with a significant diminution of the ulcerated area or total healing.

#### Safety—Dose Confirmation

The minimum dose required to cover a maximum ulcer surface area of 60 cm^2^ per participant was 0.1mL/cm^2^. On average, 1.67 (SD 0.13) sealant kits were used per participant per dressing change during the follow-up period (a minimum of 1 kit and a maximum of 3 kits per participant per dressing change).

### Secondary Outcomes

#### Preliminary Efficacy—Evaluation of the Healing Process

**Figure 4:**
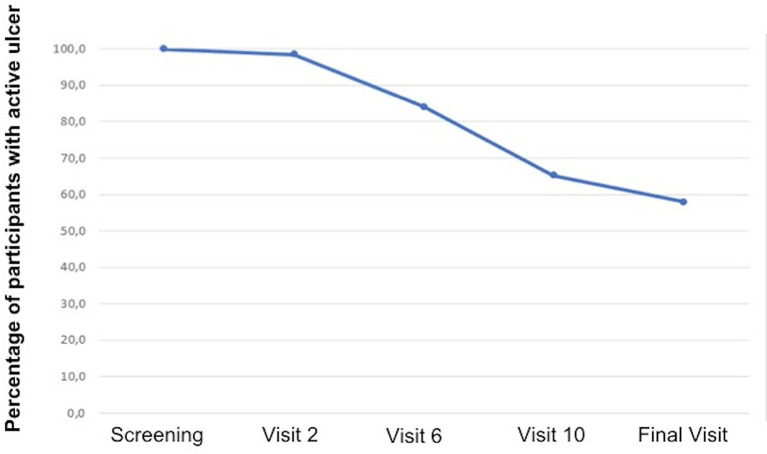
Survival analysis in relation to healing.

**Figure 5:**
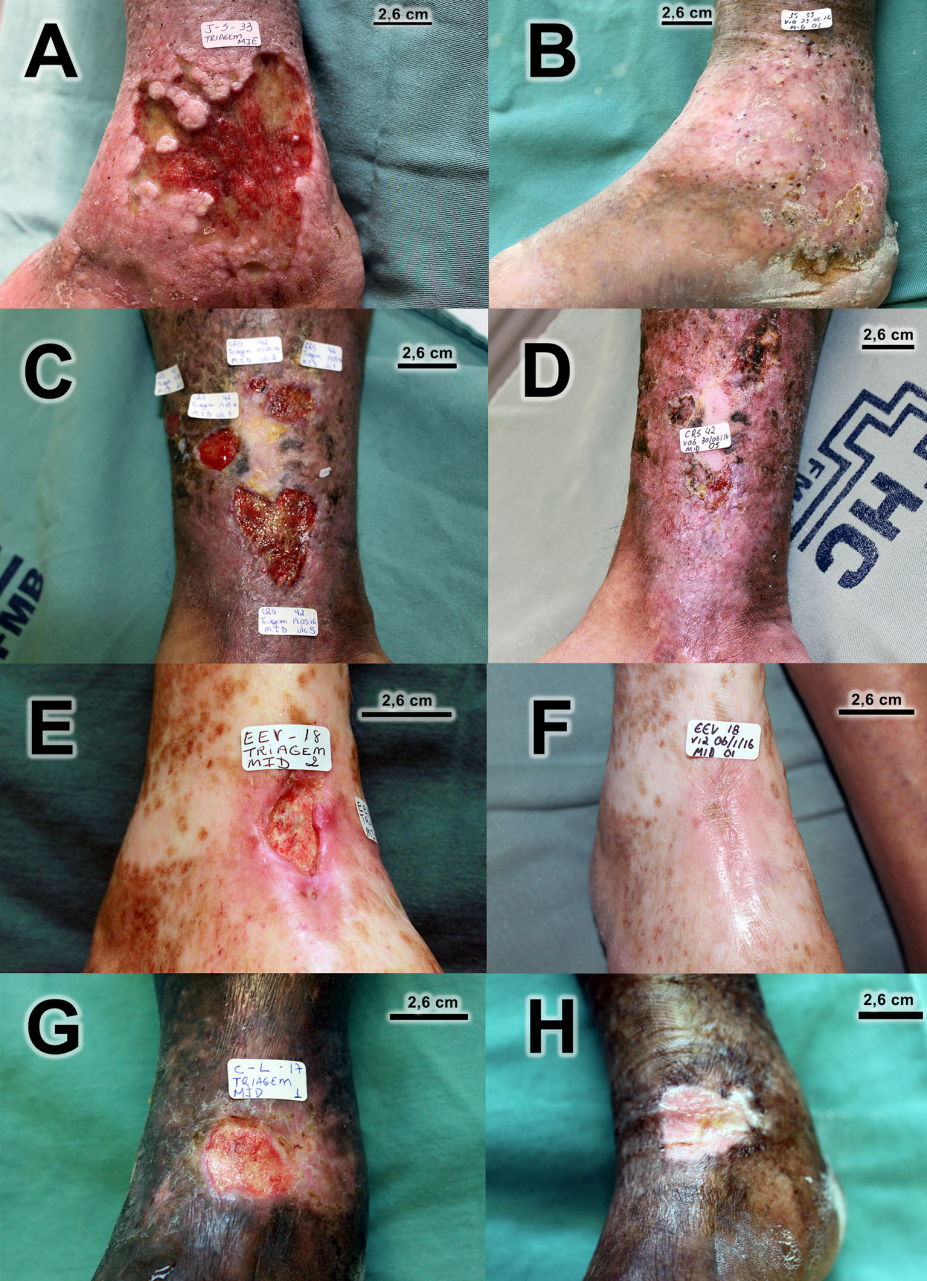
Photographic records of venous ulcers of some participants who have a good evolution of the healing process. **(A)** Before treatment (ulcer area 51,83 cm^2^)/**(B)** Complete healing at visit 10. **(C)** Before treatment (total ulcer area 19.67 cm^2^)/**(D)** Only one ulcer did not heal completely at final visit. **(E)** Before treatment (ulcer area 3.48 cm^2^)/**(F)** Complete healing at final visit. **(G)** Before treatment (ulcer area 4.89 cm^2^)/**(H)** Complete healing at visit 10.

There was healing of 28 out of the 59 ulcers that were followed up until the final visit (47.5%). **Figure 4** displays the survival curve in relation to healing. Note that after visit 2, it was already possible to start the healing process, but better results were achieved from visit 6. In addition to total healing, 13 out of 59 (22.0%) ulcers followed up until the final visit presented a reduction of ulcerated area of at least 50%. There was an improvement in the quality of the ulcerated bed when compared to the ulcers at the initial and final visits, with statistically significant differences. The quantity of exudation did not differ statistically between the initial and final visits. **Figure 5** has photographic records of some participants who have a good evolution of the healing process. **Supplementary Material 2** contains a table and graph with the evolution of the healing process of all participants followed up until to the end of the study.

## Discussion

According to the International Council for Harmonisation of Technical Requirements for Pharmaceuticals for Human Use from European Medicines Agency [ICH guideline E8 (R1)—draft version 2019], “clinical studies should be designed, conducted, and analyzed according to sound scientific principles to achieve their objectives, and should be reported appropriately. The primary objective of any study should be clear and explicitly stated” (34).

Thus, the trials were based on phases. Phase I is characterized by the initial administration of a new experimental drug in humans. It is usually conducted in healthy volunteers to assess human pharmacology (pharmacokinetics and pharmacodynamics), but also initial safety and tolerability. Phase II has the main objective of exploring therapeutic efficacy in patients. At this phase, research can use a variety of study designs, including concurrent controls and comparisons with the initial state. Phase III is designed to confirm the preliminary evidence, accumulated in phase II, that a drug is safe and effective. Therefore, its main objective is to demonstrate or confirm the therapeutic benefits, and phase IV are all studies (other than routine surveillance) performed after drug approval by the regulatory agencies (35).

The Heterologous Fibrin Sealant (HFS), a uniquely Brazilian biopharmaceutical, was already been approved in preclinical tests carried out over 20 years ago by the CEVAP team (16, 17, 36, 37). It has also been academically approved for several clinical applications, including the treatment of venous ulcers (22, 23), although without the consent of the Brazilian National Health Surveillance Agency (ANVISA). In this case, the topical use in healthy individuals is not applicable, since there is a need for patients with chronic CVUs for testing. Thus, we opted for a clinical trial called phase I/II, non-comparative, under strict control measures, to assess the safety and immunogenicity, confirm the lowest dose used, and assess the preliminary efficacy of the product in a small number of volunteers who met the inclusion criteria.

The main outcomes investigated were local and systemic adverse events, both clinical and laboratory. Among the local AEs possibly related to the product, there was mainly pain in the ulcer region, maceration and itching of the peri ulcer skin, critical colonization, peri ulcerous eczema, opening of a new ulcer, and an increase in the ulcerated area. Only one participant had pain classified as severe AE, while all others were classified as mild or moderate. It should be emphasized that no local AE has been described as definitely related to the investigational product.

To ascertain laboratory safety, particularly the possible AEs of the thrombin-like enzyme extracted from the venom of *Crotalus durissus terrificus*, the blood coagulation system of the participants was evaluated. According to the medical literature, envenomation caused by these snakes triggers important changes in blood coagulation through the consumption of coagulation factors, mainly fibrinogen and platelets (38–41). In the present study, there were no significant alterations in the levels of platelets, fibrinogen, or the erythrocyte sedimentation rate (ESR) compared to the evaluations made before, during, and at the end of treatment.

Furthermore, to assess the effect on blood coagulation, the APTT and PT of the participants were measured. The APTT is a subsidiary exam that evaluates the efficiency of the intrinsic coagulation pathway and the PT and its derivative international normalized index, also known as the international normalized ratio (INR). These are laboratory measures used to assess the extrinsic pathway of blood clotting. These hematological parameters remained stable and without significant changes when compared before, during, and at the end of treatment. The laboratory findings allowed us to conclude that no participant had a clinical manifestation of blood dyscrasia.

The product’s laboratory safety was also assessed by triggering the acute phase reaction, which could be caused by the various proteins present in the product’s components. This reaction was evaluated by alterations in the levels of white blood cells and C-reactive protein (CRP), a specific marker of acute phase reaction in humans (42–44). The observed results indicated that this reaction was not triggered. Therefore, it was possible to conclude that there was no systemic AE related to the product from the laboratory point of view.

The evaluation of immunogenicity was mainly based on the Guidelines of the European Medicines Agency—Science Medicine Health (45) and on the Guidance for Industry from the U.S. Department of Health and Human Services of the Food and Drug Administration (46). According to these guidelines, most biological and biotechnology-derived proteins induce an immune response. The immune response in these cases varies from individual to individual and involves several factors, including genetics, age, the disease to be treated, the proposed dosage, the route of administration, the presence of antibodies due to previous exposure to similar products, and the patient’s own sensitivity (24, 47). The IgA, IgE, IgG, and IgM immunoglobulins were measured prior to and during treatment. The development of immunogenicity caused by products of biological origin may directly interfere with the efficacy and safety of the product under investigation and may even hinder the continuation of clinical trials. In this case, in addition to the detailed clinical analysis of the participants, one must seek, through biochemical methods, to produce neutralizing (IgM and IgG) and non-neutralizing (IgA and IgE) antibodies before and during treatment. Neutralizers can cause a loss in the product’s effectiveness by binding strongly to the active sites of the drug. Non-neutralizers, in general, trigger hypersensitivity reactions, although they may decrease the product’s effectiveness indirectly. The latter, in most cases, is of the IgE class and may induce, due to hypersensitization, a reaction denominated as Coombs and Gell type I (48). This ranges from simple rhinitis, fever, asthma, and eczema to the severe form of the anaphylactic reaction (49–51).

In the present study, no patient presented a change in IgM levels before, during, or after treatment. Therefore, the investigational product did not interfere with the acute immune response. As for IgG and IgA, no participant showed significantly altered levels in relation to the reference values. Those levels above the reference values were found to be unrelated to any immunogenic reaction caused by the product under investigation because some dosages were already above and diminished throughout the study or remained high even before starting treatment.

Many participants in our study demonstrated altered total IgE levels in relation to the reference values. However, it should be emphasized that several patients possessed high levels of this antibody before starting treatment. To complement this evaluation, we performed dot blots to check for the presence of specific type E immunoglobulins; that is, an assay that can determine whether the change in the amount of IgE is directly related to the patient’s exposure to the two main components of the product (52). In relation to cryoprecipitate, four participants were positive at the end of treatment. Of these four, two were negative before and positive at the end, and two were already positive and remained positive at the end of the treatment. Coincidentally, both also presented elevated levels of IgE through the nephelometry test before the start of treatment, and therefore, prior to exposure to the bioproduct. It should be emphasized that these patients, despite the alterations observed, had a good evolution of the healing process. Therefore, variations in serum IgE levels were not related to the investigational product. As for the thrombin-like enzyme, no patient had a positive reaction either before or at the end of treatment. In this case, the results suggest that this molecule was not able to sensitize the participants. It is important to note that other reasons may account for the elevated IgE levels in these participants, such as parasitic diseases, viral infections, immunological diseases, neoplasms, or hepatitis. Furthermore, factors such as sex, race, smoking status, season, and genetic potential can interfere with IgE synthesis (51).

Therefore, regarding the immunogenicity analyses, considering the four antibodies evaluated combined with anamnesis of the participants (background and history prior to the study) and the physical examinations performed during the visits, no participant presented clinical symptoms of hypersensitivity to the 12 applications of the product during the three months of treatment. It is concluded, therefore, that despite the application of the investigational product to the skin, which is not only the largest organ in the human body but also possesses greater capacity for hypersensitization (53), HFS did not show immunogenicity to their components.

Although the study was designed to primarily assess the safety of the product, we evaluated the healing outcomes for a preliminary analysis of efficacy. The results indicated a promising efficacy with an average application dose of 1.6 sealant kits per participant, per dressing change, in the proposed time period. There was healing of 47.5% of the ulcers, while 22% showed a significant reduction in the area, in addition to a qualitative improvement of the ulcerated bed of the unhealed ones. The healing capacity of the product will need to be confirmed through a phase III study that will be a multicentric, randomized controlled, and double-arm trial with a sample size sufficient to provide statistical power, resolvability, and reproducibility.

The fact that the study is of a non-randomized arm, without a comparative group, does not make it a limiting factor, since its main objective was to assess safety in relation to the dose chosen for the experimental product. As it is a bioproduct, an evaluation of the results of immunogenicity is indispensable, as the bioproducts are very immunogenic, which often prevents its continuous and long-term use, thus limiting its application. Immunogenicity was not verified in this study for at least 12 weeks.

Although it was possible to verify the assessed time, complete healing of 47.5% of chronic venous ulcers, a randomized, double-arm phase III study should be performed to corroborate the effectiveness. Likewise, some AEs may have occurred regardless of the investigational product due to the influence of other confounding factors, such as concomitant medications, the evolution of the disease, the care of the patients with the wounds, and genetic factors, including unknown factors (54). For this reason, there is a need to prove these results with a randomized controlled trial with a comparative group with standard treatment for CVUs.

It is important to emphasize that we evaluated fewer patients than registered in our protocol. The sample size was estimated considering examples of phase I/II clinical studies, which aim to study safety. In our original protocol, we recorded that it would be carried out in 40 participants, but as this study was a single-arm study and the main objective was safety, there was no formal calculation of the sample size. Therefore, when we reached the inclusion of 31, we concluded that there were enough data to finalize the study.

The biggest challenges of our study were as follows: first, to prospect, produce, and develop a new toxin-based bioproduct within good laboratory practices, then convince the regulatory agency and the ethics committee to authorize the conduct of the clinical trial; and second, to convince patients to join the study. As most of the candidate molecules found in animal toxins are present in very low amounts, isolation and production was a major challenge, and was only possible due to the development of the technical methodologies for the isolation and purification of the components used, allowing for its future registration and commercial scaling (12, 15, 24, 26).

## Conclusions

The investigational product heterologous fibrin sealant (HFS) proved to be safe for the treatment of CVUs according to the proposed dosages. There were no systemic AEs related to the product, whereas the few local AEs were mild to moderate in intensity. Regarding immunogenicity, it was observed that neither neutralizing (IgM and IgG) nor non-neutralizing antibodies (IgA and IgE) were produced or presented significant deviations. As for preliminary efficacy, there was total healing in 47.5% of the ulcers. For those that did not heal, there was a significant reduction in the area by 22%, in addition to an improvement in the quality of the ulcerated bed. Therefore, the investigated product was demonstrated to be safe and non-immunogenic, with promising efficacy for the treatment of CVUs in the proposed dosages. A multicentric phase III clinical trial will be needed to verify these findings.

## Data Availability Statement

The original contributions presented in the study are included in the article/**Supplementary Material**. Further inquiries can be directed to the corresponding authors.

## Ethics Statement

The studies involving human participants were reviewed and approved by the National Commission on Ethics in Research (CONEP, Certificate of Presentation of Ethical Appreciation No. 37410814.4.0000.5411, v8, approved on 12/16/2014). The patients/participants provided their written informed consent to participate in this study.

## Author Contributions

LA, BB, and RF contributed to the conception and design of the study. LA, NM, and MC contributed to the study planning and design of the electronic form (Electronic Case Report Form—eCRF), which was developed specifically for this study. SB, MS, AL, GH, and MG contributed to the clinical data collection. LS was responsible for the immunobiological analyses. LA and BB performed the data analysis and drafted the manuscript. All authors contributed to the article and approved the submitted version.

## Funding

This study was supported by the National Council for Scientific and Technological Development, CNPq, Proc. No. 563582/2010-3 (BB), CNPq Proc. No. 458919/2014-4 (LS), and CNPq Proc. No. 401170/2013-6 (BB); São Paulo Research Foundation, FAPESP, Proc. No. 2014/13299-7 (LS); São Paulo Research Foundation, FAPESP, Proc. No. 2021/00451-9 (LPFA); the Coordination for the Improvement of Higher Education Personnel, CAPES, through Toxinology CAPES Call No. 063/2010, Proc. No. 23038.006285/2011-21, AUXPE Toxinology 1219 (BB). RF is a CNPq PQ1C research fellow No. 303224/2018-5.

## Conflict of Interest

The authors declare that the research was conducted in the absence of any commercial or financial relationships that could be construed as a potential conflict of interest.
