# Supplementary material for: Treatment of Chronic Venous Ulcers With Heterologous Fibrin Sealant: A Phase I/II Clinical Trial
Source: Front Immunol. 2021 Feb 23;12:627541. doi: 10.3389/fimmu.2021.627541 (PMC7940668; doi:10.3389/fimmu.2021.627541)
Supplement: Supplementary file 1 [file DataSheet_1.pdf]

Supplementary material 1- Laboratory tests.

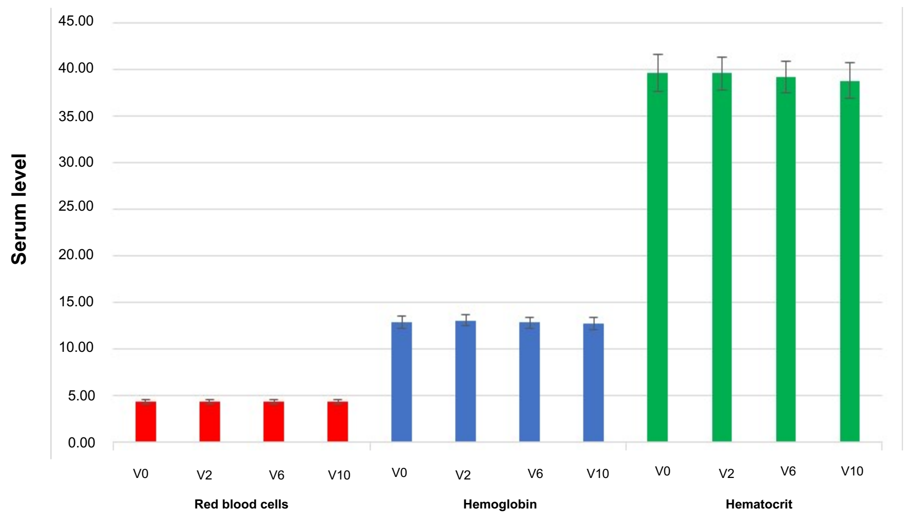

Figure 1: Mean serum levels of red blood cells, hemoglobin and hematocrit, according to visits, with their respective 95% confidence intervals. V0: screening; V2: visit 2; V6: visit 6; V10: visit 10. Reference values - red blood cells: 4.20 to 5.40 million / mm^3; Hemoglobin: 12.00 to 16.00 g / dL; Hematocrit: 37.00 to 47.00%.

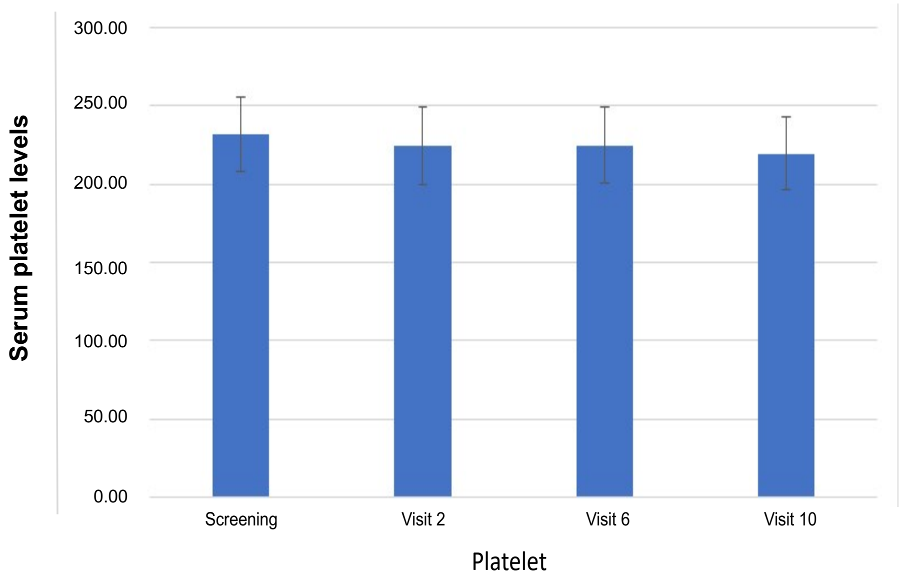

Figure 2: Mean serum platelet levels according to visits, with their respective 95% confidence intervals. Reference values: 140.00 to 440.00 $x10^3$ / mm^3.

.

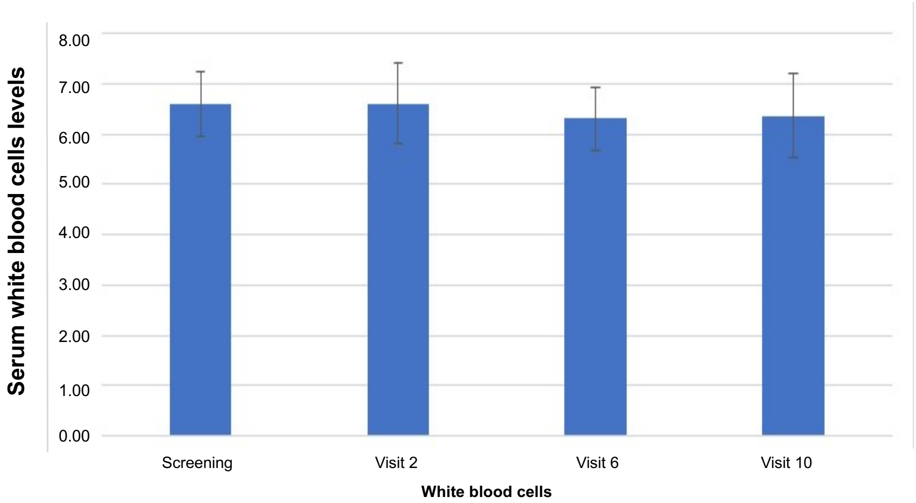

Figure 3: Mean serum white blood cells levels according to visits, with their respective 95% confidence intervals. Reference values: 4.00 to 11.00 x10^3 / mm^3.

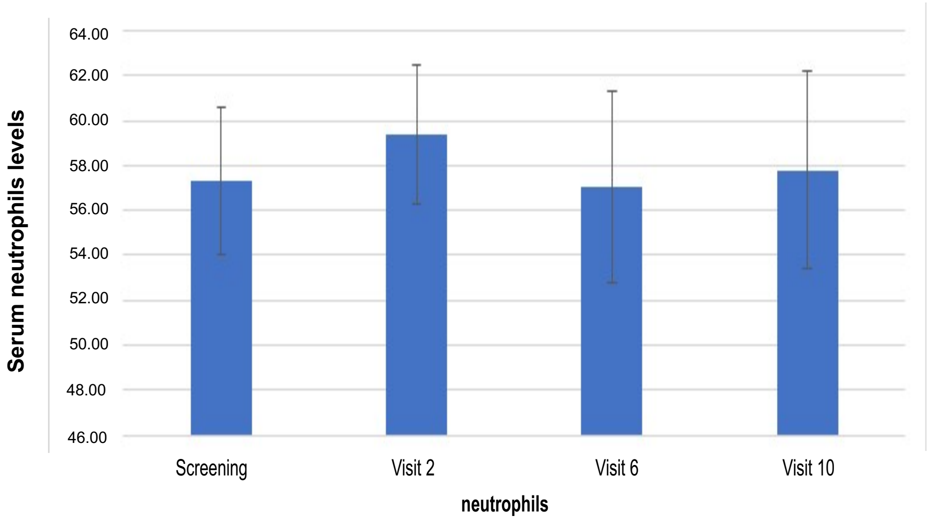

Figure 4: Mean serum neutrophils levels according to visits, with their respective 95% confidence intervals. Reference values: 53.00 to 67.00%.

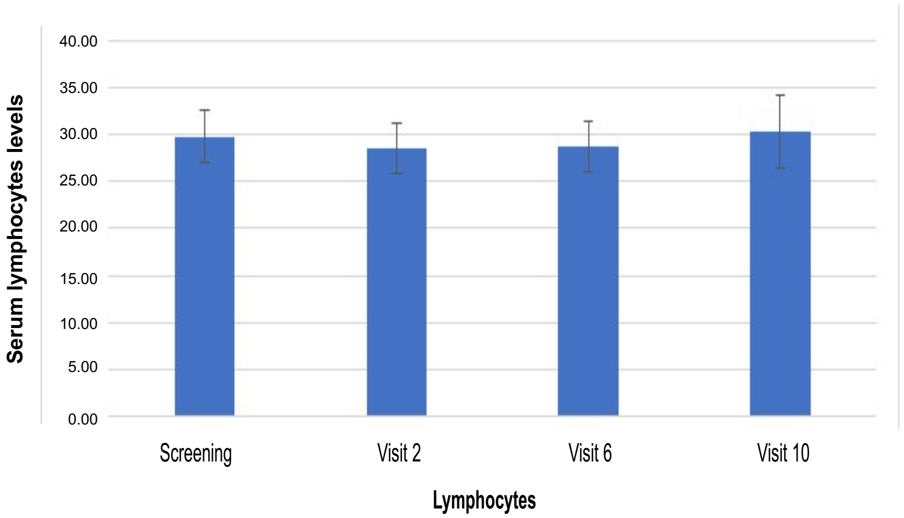

Figure 5: Mean serum lymphocytes levels according to visits, with their respective 95% confidence intervals. Reference values: 23.00 to 33.00%.

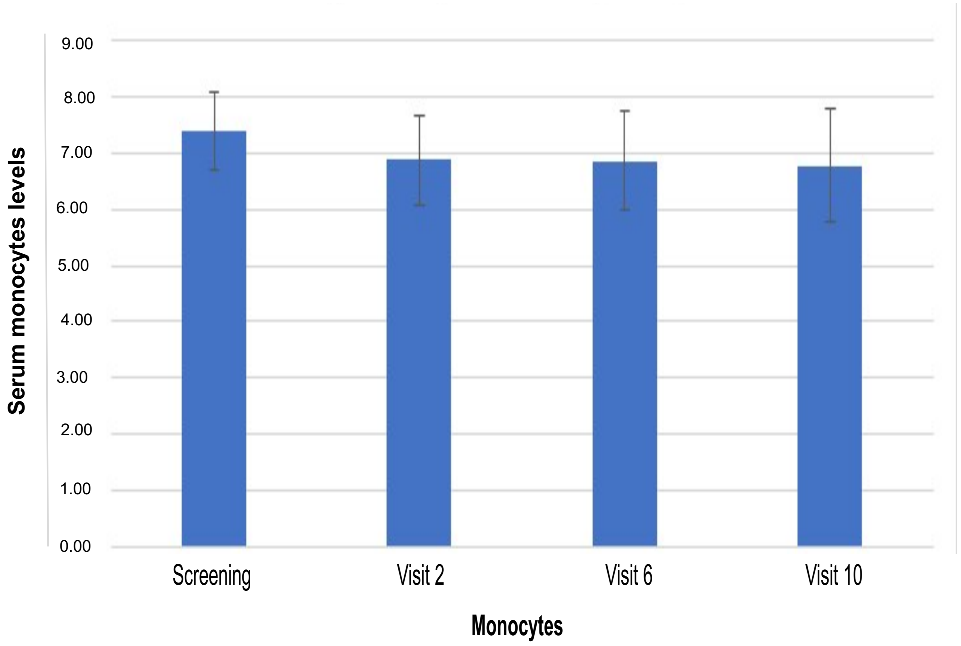

Figure 6: Mean serum monocytes levels according to visits, with their respective 95% confidence intervals. Reference values: 3.00 to 8.00%.

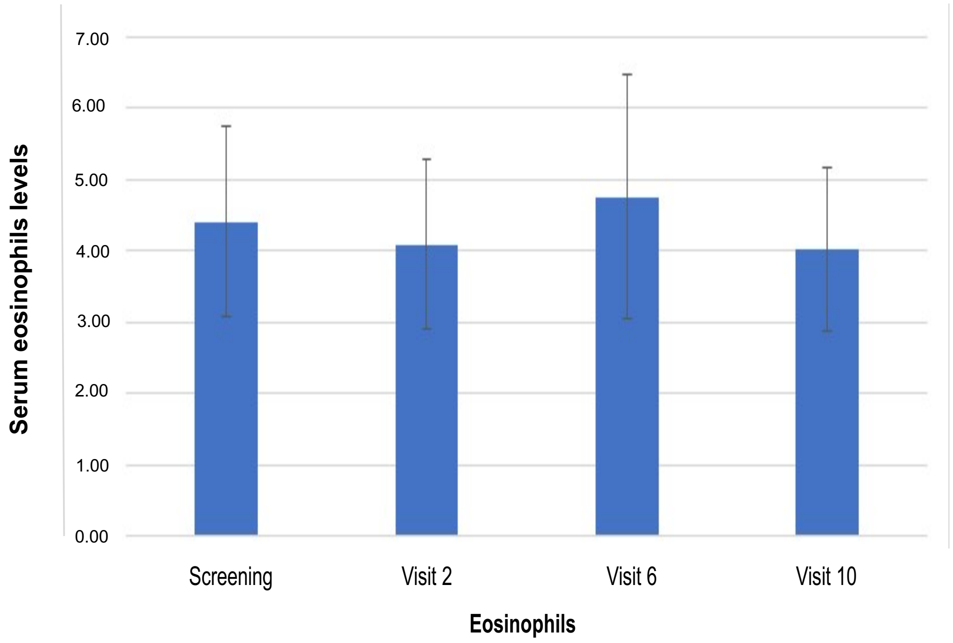

Figure 7: Mean serum eosinophils levels according to visits, with their respective 95% confidence intervals. Reference values: 2.00 to 4.00%.

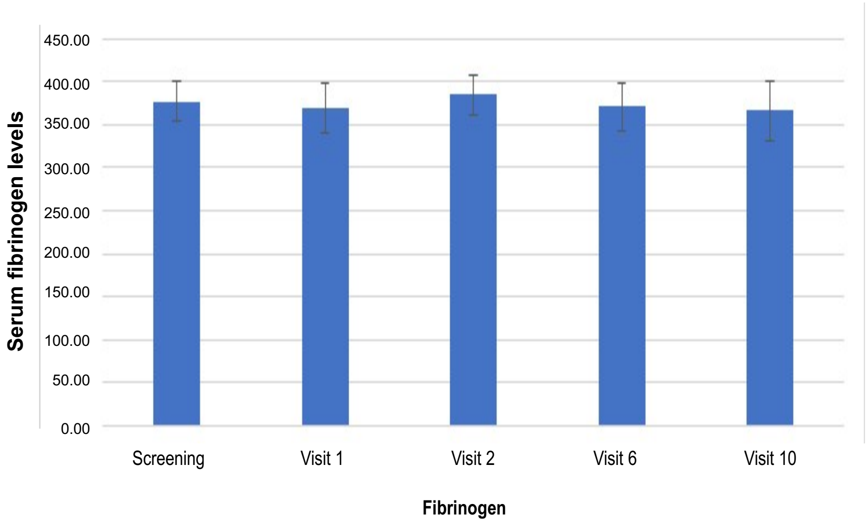

Figure 8: Mean serum fibrinogen levels according to visits, with their respective 95% confidence intervals. Reference values: 146.00 to 380.00 mg / dL

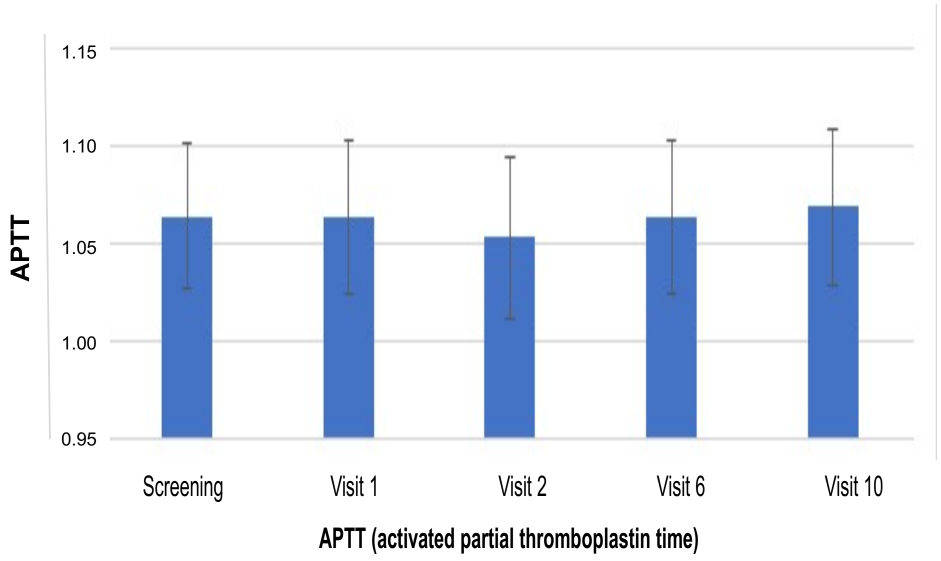

Figure 9: Mean value of APTT (activated partial thromboplastin time) according to visits, with their respective 95% confidence intervals. Reference value: up to 1.25 for non-anticoagulated patients.

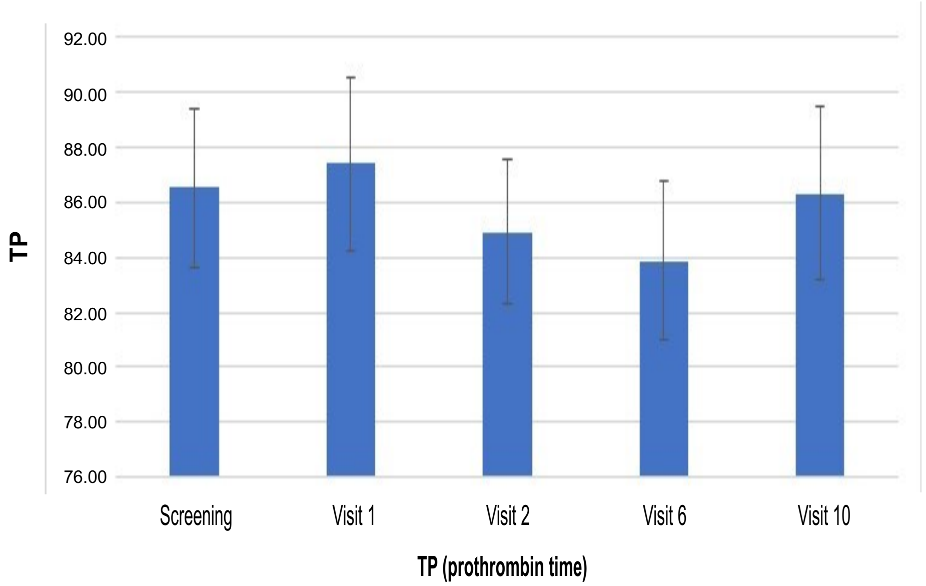

Figure 10: Mean value of TP (prothrombin time) according to visits, with their respective 95% confidence intervals. Reference value: 70.00 to 100.00%.

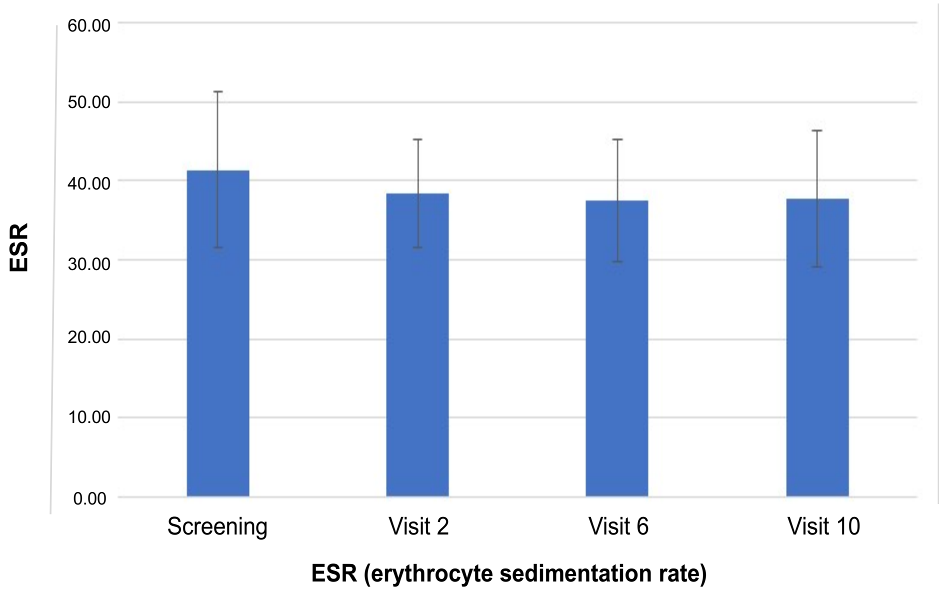

Figure 11: Mean value of ESR (erythrocyte sedimentation rate) according to visits, with their respective 95% confidence intervals. Reference values: Men up to 10.00 mm / h and women up to 20.00 mm / h.

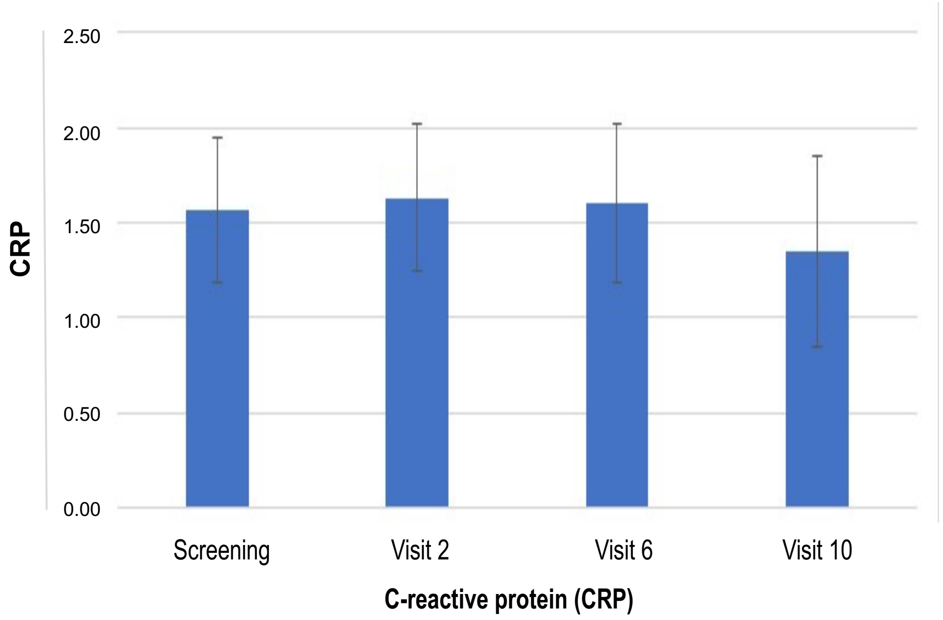

Figure 12: Mean value of C-reactive protein (CRP) according to visits, with their respective 95% confidence intervals. Reference values: <1.00 mg /dl.
