# Supplementary material for: Treatment of Chronic Venous Ulcers With Heterologous Fibrin Sealant: A Phase I/II Clinical Trial
Source: Front Immunol. 2021 Feb 23;12:627541. doi: 10.3389/fimmu.2021.627541 (PMC7940668; doi:10.3389/fimmu.2021.627541)
Supplement: Supplementary file 2 [file DataSheet_2.pdf]

Supplementary Material 2 – Evolution of the healing process

Tabela 1: Data related to the healing process of 25 participants (59 ulcers) who were followed up until the end of the study.

Participant	Number of ulcer per participant (n=59)	Initial ulcer area	Final ulcer area
1	1*	10.89	0.0
	2*	6.38	0.0
2	1*	1.94	0.0
	2†	13.10	3.23
3	1#	2.48	13.02
	2#	2.40	6.8
	3#	4.66	10.75
4	1#	21.65	39.45
	2#	31.25	43.53
5	1*	4.08	0.0
	2*	0.26	0.0
	3*	1.60	0.0
	4†	3.67	1.31
6	1†	2.23	1.24
	2†	5.37	0.26
7	1*	6.17	0.0
	2†	4.87	1.82
8	1*	0.41	0.0

	2*	3.58	0.0
9	1*	1.90	0.0
	2*	1.10	0.0
	3*	1.30	0.0
	4†	31.10	5.3
	5*	2.70	0.0
	6*	0.40	0.0
10	1#	2.86	13.05
	2#	12.68	16.84
11	1#	36.52	68.99
	2#	2.48	5.67
	3#	5.37	6.88
12	1#	41.14	91.48
	2*	5.84	0.0
	3*	11.47	0.0
13	1*	11.19	0.0
14	1†	36.43	5.55
15	1†	60.00	50.26
16	1†	14.87	4.70
17	1†	17.39	8.75
18	1†	1.67	0.62
	2*	1.49	0.0
	3*	0.85	0.0
	4*	0.35	0.0
	5†	17.63	2.28

19	1*	2.94	0.0
	2*	0.23	0.0
	3*	2.66	0.0
	4*	1.50	0.0
	5*	12.34	0.0
20	1*	2.93	0.0
21	1†	8.09	4.49
	2†	3.60	3.29
	3*	1.85	0.0
	4#	15.23	17.22
22	1†	6.36	5.12
23	1†	0.29	0.81
	2†	4.11	1.06
24	1†	9.25	0.77
	2†	31.90	10.97
25	1*	51.83	0.0

* Ulcers healded

† Reduction of the ulcerated area

Increased of the ulcerated area

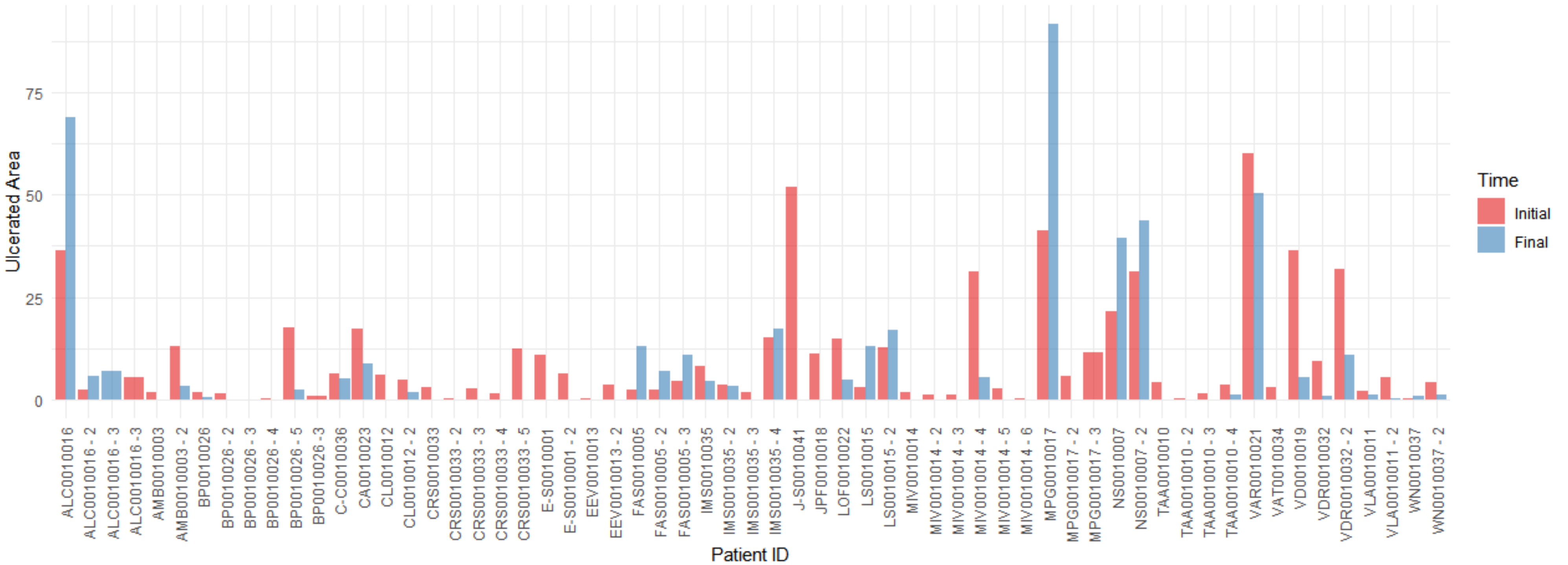

Figure 1: Comparasion between initial and final ulcerated area per ulcer of 25 patients (59 ulcers).
